# Hepatitis C virus modified _S_E2_F442NYT_-mRNA-LNP candidate vaccine promotes helper CXCR5^+^T cells

**DOI:** 10.1128/jvi.01355-25

**Published:** 2025-09-05

**Authors:** Yuki Haga, Preedia Babu E, Marzena Swiderska-Syn, Erin K. Reagan, Drew Weissman, Ranjit Ray

**Affiliations:** 1 Department of Internal Medicine, Saint Louis University 7547, St. Louis, Missouri, USA; 2 Department of Paediatrics, Saint Louis University 7547, St. Louis, Missouri, USA; 3 Department of Medicine, University of Pennsylvania 6572, Philadelphia, Pennsylvania, USA; Wake Forest University School of Medicine, Winston-Salem, North Carolina, USA

**Keywords:** HCV, sE2, sE2_F442NYT_, NK cells, DCs, follicular B helper T cells, Th17 cells

## Abstract

T cells play an important role in initiating antibody responses by instructive signals of cell-cell contacts and secretion of soluble cytokines as mediators. We investigated the role of the modified soluble E2 (sE2_F442NYT_) antigen from hepatitis C virus (HCV) on healthy human peripheral blood mononuclear cell (PBMC)-derived immune cells or immunized mouse cells to understand the mechanisms of immune regulation by the candidate vaccine antigen. HCV E2 and E2_F442NYT_ displayed a role in inducing type 17 T-helper cell (Th17) phenotype, as indicated by interleukin-17 (IL-17) expression and signal transducer and activator of transcription 3 (Stat3) phosphorylation. The spleen cells from sE2-mRNA-lipid nanoparticles (LNPs) or sE2_F442NYT_-mRNA-LNP-immunized mice exhibited similar IL-17A mRNA levels, and Th17 (CXCR3^−^CCR6^+^) cells in CD4^+^CD44^+^ spleen cells, supporting both sE2 and modified sE2_F442NYT^-^_induced Th17 polarization. Immunohistochemical and multiplex immunofluorescence imaging studies revealed abundant CD4^+^CXCR5^+^T cells co-localized with BCL6 in sE2_F442NYT_-mRNA-LNP immunized mouse spleen cells than unmodified sE2-mRNA-LNP immunized animals, suggesting sE2_F442NYT_ induces stronger follicular helper T cell generation. We previously demonstrated increased total IgG production and isotype switching from IgG1 to IgG2a and IgG2b in sE2_442NYT_ immunized mice. The stronger B and T cell responses observed from modified sE2_F442NYT_ support the overall *in vivo* outcome of the study toward a higher B helper T cell generation from sE2_F442NYT_-mRNA-LNP immunization as compared to unmodified sE2-mRNA-LNP.

**IMPORTANCE:**

The study will help rationalize HCV vaccine antigen selection for an effective immune response. Extension by additional strategies may be useful to direct stronger B helper T cell generation for prolonged vaccine-associated protection.

## INTRODUCTION

HCV infection often progresses to a chronic state and is a leading cause of global liver disease. Interferon lambda (IFNλ4) regulates host response to hepatitis C virus (HCV) infection. A single nucleotide polymorphism (SNP) in the interferon lambda 4 gene was linked to an increased probability of spontaneous viral clearance and sustained virological response (1). IFNλ4, a genetic variant of IFNλ, is identified as the molecular link between the various genetic variants involved in spontaneous and treatment-induced HCV clearance leading to the so called IFNλ4 paradox. IFNλ4 is shown to have a negative impact on antigen-dependent immune responses that could explain the “IFNλ4 paradox.” IFNλ4-induced ER stress impairs HCV antigen processing and/or loading onto the MHC I complex. Consequently, T cell responses are attenuated, favoring viral escape from the cellular immune response affecting viral clearance (2).

Recent studies suggested spontaneous HCV clearance requires strong, sustained, and multiepitope-specific immune responses from CD4^+^T, CD8^+^T, and B cells. These responses are crucial for controlling and eliminating the virus from the body (3, 4). To date, no prophylactic HCV vaccine candidates showed satisfactory results in the induction of effective immune response or preventing chronic infection.

We previously conducted a Phase I vaccine trial of recombinant HCV E1/E2 EnvGPs:MF59 in healthy human volunteers (5–8). HCV E1/E2 envelope glycoprotein vaccination induced a weak T- and B-cell responses in healthy adults. The antibody response to HCV E1/E2 in vaccinated sera was primarily IgG1 subclass. Antibody avidity index was established after the third dose of immunogen and was not affected by the dosage level delivered during vaccination. Only 10 of 36 vaccinated sera had a neutralization titer of ≥1:20 against cell culture-grown HCV genotype 1a. Neutralizing sera had increased affinity levels and displayed >2-fold higher specific activity to well-characterized epitopes, especially to the hyper variable region 1 (HVR1) of E2 and multiple genotype-specific neutralizing epitopes of E1 and E2 (9, 10). A different vaccine-related study using live adeno virus vector approach to induce T-cell responses to HCV non-structural region did not protect intravenous drug users from HCV infection (11). One possible explanation could be for not including HCV envelope glycoprotein, which are the primary targets of neutralizing antibodies. The persistence of infection may be due to viral escape mechanism under immune pressure and limited cross- reactivity of vaccine-induced T cell to HCV (11).

A limited immune response in recipients has been attributed to several factors, including HCV’s vast genetic and antigenic diversity and significant structural flexibility within epitopes. In addition, we recently observed an induction of interleukin-10 (IL-10) and signal transducer and activator of transcription 3 (Stat3) activation in human macrophages incubated with recombinant E2 glycoprotein with CD81 binding activity, suggesting macrophage polarization toward the M2 phenotype (12). Furthermore, we observed HCV E2 exhibits immunoregulatory activity inhibiting the induction of a robust protective immune response, such as Th2 cell polarized cytokine response and induction of a weaker neutralizing antibody response, in vaccinated mice (13), suggesting that an immunoregulatory role of parental E2 or transmembrane-deleted soluble E2 (sE2) on protective immune response-related cells. Complex structural make-up of proteins, including intrinsically disordered HCV envelope glycoproteins, should be considered for their role in the regulation of host defense mechanisms (14). We recently modified the sE2 sequence at a CD81 receptor-binding site with point mutations and inclusion of a novel glycosylation site (13). The modified sE2_F442NYT_ had an impaired CD81 interaction. Immunization with sE2_F442NYT_-mRNA-LNP exhibited altered cytokine parameters associated with an improved proinflammatory response, enhanced T cell response, antibody class switch recombination (CSR), and elevation of cross-genotype neutralizing antibody response, resulting in robust protection against challenge infection with surrogate HCV recombinant vaccinia virus in mice (13, 15–17). Subsequently, we reported that the stimulation by unmodified HCV sE2 suppressed activation-induced cytidine deaminase (AID) and basic leucine zipper ATF-like transcription factor (BATF) proteins, as one of the underlying mechanisms for the regulation of antibody CSR transcripts (16, 17).

In this study, we have examined whether sE2_F442NYT_ induces Th17 cells and follicular helper T (Tfh) cells in promoting B cell differentiation and CSR. Our findings suggest sE2_F442NYT_ induces helper CXCR5^+^T cell generation and contributes as an effective HCV vaccine antigen for induction of protective immune response.

## RESULTS

### sE2_F442NYT_ induces IL-6 in monocyte-derived DCs

**Fig 1:**
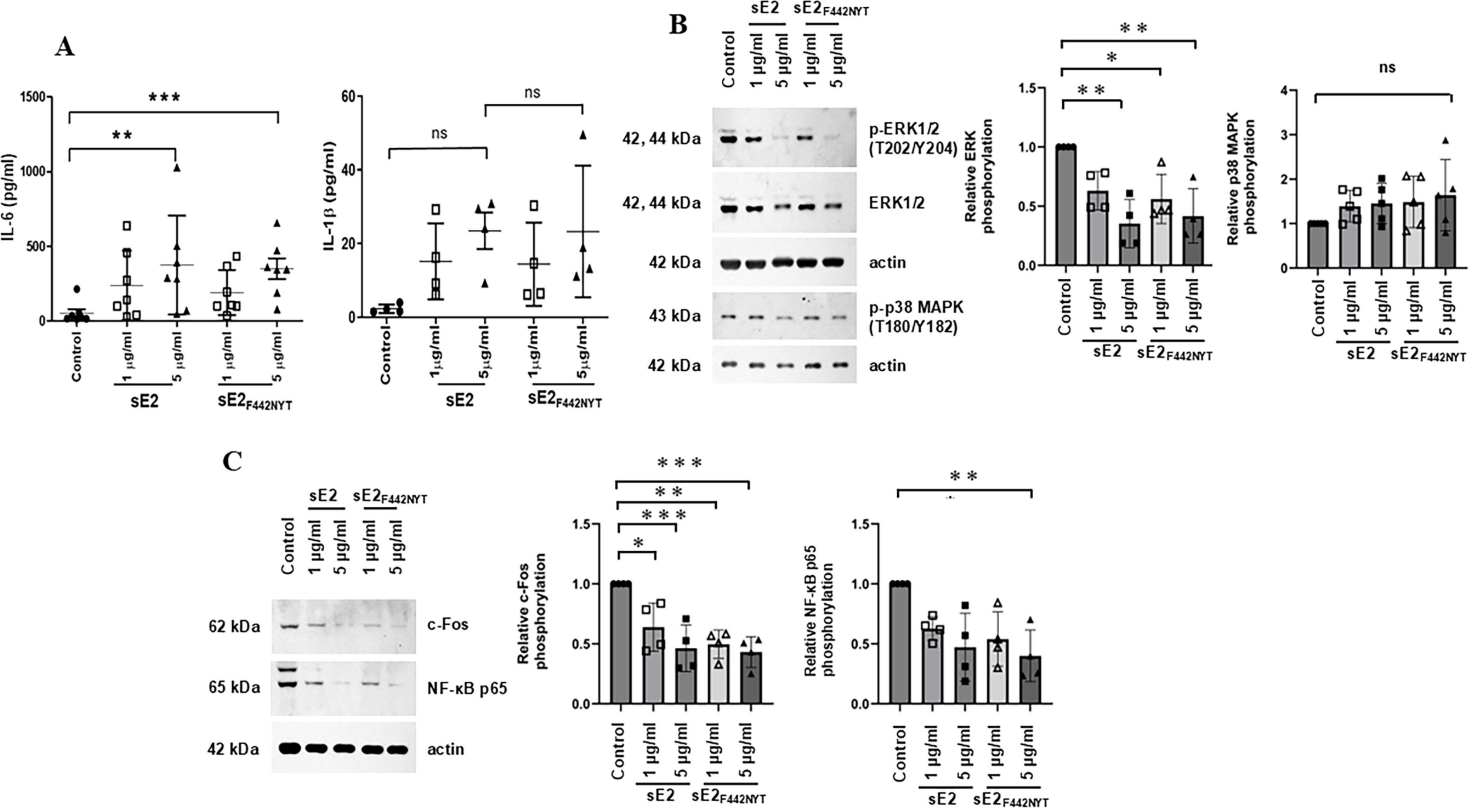
sE2 and modified sE2_F442NYT_ enhance IL-6 production in human dendritic cells (DCs). (**A**) IL-6 (*n* = 7) and IL-1β (*n* = 4) expression in monocyte-derived DCs incubated with purified sE2 or modified sE2_F442NYT_ proteins. Data are presented as mean ± SD. (**B**) Western blot analysis of ERK1/2 and p38 MAPK phosphorylation in monocyte-derived DCs. (**C**) Western blot analysis of c-Fos and NF-κB p65 expression in monocyte-derived DCs. Representative blots are shown, with densitometry scanning results presented as bar graphs. Values are expressed as mean ± SD, normalized to actin content and relative to controls (set at 1.0). Statistical significance was determined by one-way ANOVA and marked as *P < 0.05, **P < 0.01, ***P < 0.001 or *P* > 0.05 (ns).

IL-6 is one of the contributing factors for the development of defined effector populations, like Th17 and Th22 subsets of helper T cells or their inhibition, such as regulatory T cells (Tregs) in humans. IL-6 governs the proliferation, survival, and commitment of T cells and modulates their effector cytokine production (18). IL-6 is required for the proliferation and survival of plasma blasts with the ability to produce antibodies by either directly acting on plasma cells or indirectly promoting Tfh cell differentiation (15) and induces differentiation of CD8^+^ T cells into effector CD8^+^T cells (19). Counter functional to Tregs is the Th17 cell group, which mediates the inflammatory response upon recognition of foreign antigens (20). We have reported that sE2_F442NYT_ induces higher levels of IL-6 from antigen-presenting cells (APCs) including human macrophages and B cells *in vitro* (13, 16). To evaluate whether HCV E2 antigens affect cytokine secretion from other powerful APCs, human primary monocyte-derived DCs were treated with HCV antigens. Purified sE2_F442NYT_ treatment significantly stimulated IL-6 when compared to the untreated control DCs (Fig. 1A). IL-6 increase in DCs by HCV E2 antigen was dose-dependent and similar between sE2 and sE2_F442NYT_ treatment. IL-1β is an important mediator of inflammatory responses and involves a variety of cellular activities, including cell proliferation and differentiation. IL-1β is crucial for host defense responses (21). sE2 and sE2_F442NYT_ also enhanced IL-1β production equally. On the other hand, IL-23 levels did not change significantly upon HCV sE2/sE2_F442NYT_ treatment (Fig. S1). Previously, we reported the effect of E2 on the extracellular signal-regulated kinase (ERK) phosphorylation in human primary B cells and T cells (16). ERK, also known as the mitogen-activated protein kinase (MAPK), is activated upon stimulation leading to phosphorylation of its regulatory Tyr and Thr. It plays a major role in mediating inflammatory as well as oncogenic signals. MAPK pathways have a role in mediating IL-6 production (22). ERK phosphorylation was significantly inhibited by sE2 and sE2_F442NYT_ (Fig. 1B). The level of p-38 MAPK phosphorylation did not appear to show a significant change upon sE2 or sE2_F442NYT_ treatment. The nuclear factor-kappa B (NF-*κ*B) family of transcription factors regulates the expression of a wide range of genes critical for immune and inflammatory responses, cell survival, immune development, and cell proliferation (23). NF-κB and c-Fos are transcription factors activated in immune cells and in most other cell types following stimulation by a variety of factors, including cytokines and growth factors. c-Fos was reduced by sE2 or sE2_F442NYT_ in DCs, while NF-κB was similar or decreased upon sE2 or sE2_F442NYT_ antigen stimulation (Fig. 1C). Thus, both sE2 and sE2_F442NYT_ induced IL-6, which helps in promoting inflammatory responses for protective immune regulation against HCV.

### sE2 and sE2_F442NYT_-stimulated modest IL-17 induction and Stat3 activation by DCs in autologous primary human CD4^+^T cell co-culture

**Fig 2:**
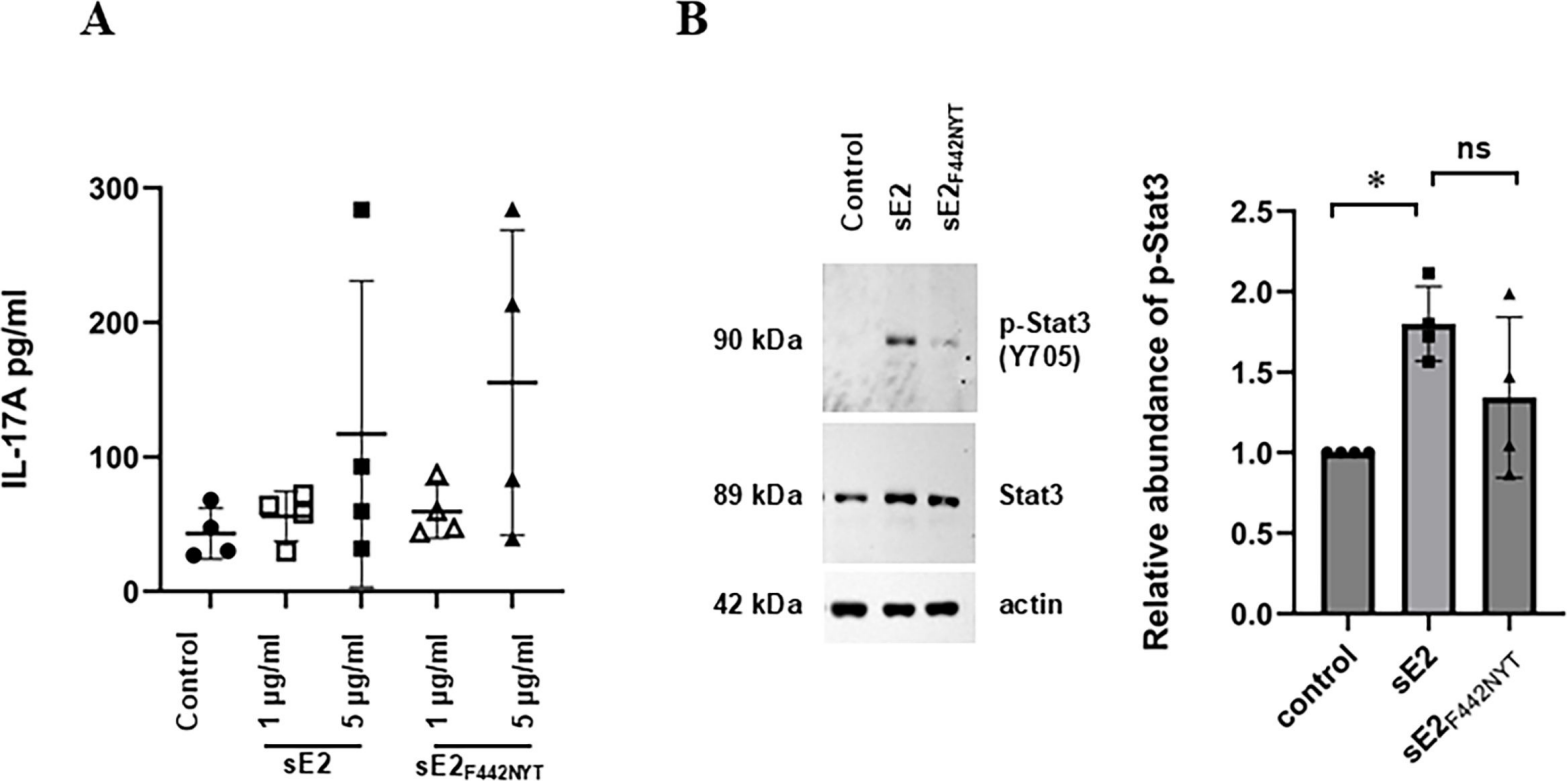
sE2 and sE2_F442NYT_-stimulated dendritic cells (DCs) enhance IL-17A production in autologous CD4^+^T cells. (**A**) IL-17A generation by CD4^+^T cells from healthy donors co-cultured with autologous monocyte-derived DCs stimulated with sE2 or modified sE2_F442NYT_ (*n* = 4). There was no statistical significance between sE2 and modified sE2_F442NYT_ treatment. (**B**) Phosphorylation of Stat3 (p-Stat3) in CD4^+^T cells, summarized from data of 4 donors, presented as bar graphs. Data are shown as mean ± SD. Statistical significance was determined by one-way ANOVA test.

IL-6 induction is linked with multiple transcription factors, such as Stat3, which promotes Th17 cell generation and IL-17 expression (24). After stimulating monocyte-derived DCs with sE2 or sE2_F442NYT_ for 24 h, we co-cultured autologous human primary CD4^+^T cells for 7 days and analyzed IL-17A levels in culture fluid by ELISA (Fig. 2A). sE2 or sE2_F442NYT_-stimulated DCs increased IL-17A generation from CD4^+^T cells, and increased Stat3 phosphorylation, a required transcription factor for early Th17 cell differentiation (25). Stat3 was phosphorylated when cells were treated with sE2 compared to untreated control, while we did not observe a significant difference between sE2 and modified sE2_F442NYT_-treated cells (Fig. 2B), suggesting both sE2 and sE2_F442NYT_ induce Th17 response.

### sE2-and sE2_F442NYT_-mRNA-LNPs induce IL-17A in immunized mouse spleen

**Fig 3:**
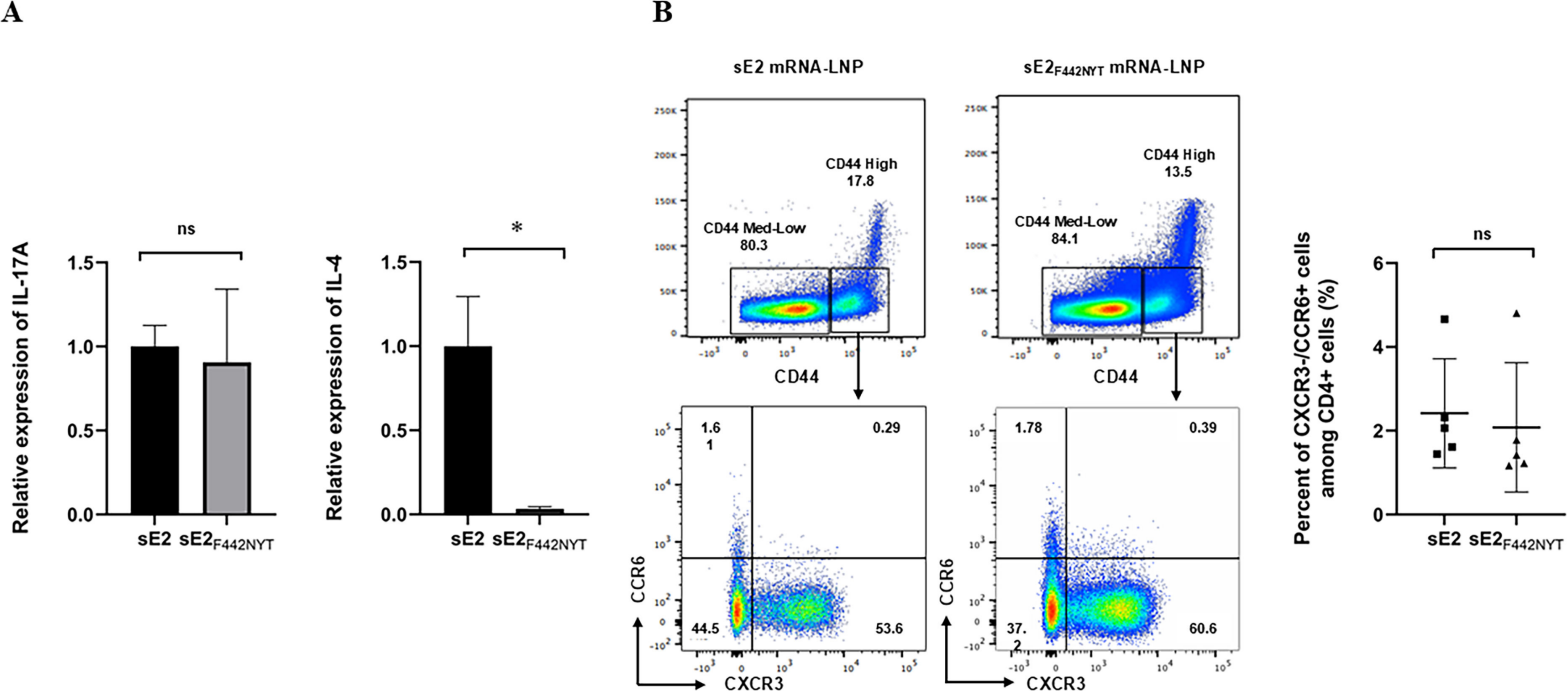
Detection of potential Th17 cell subsets in sE2 and sE2_F442NYT_-mRNA-LNP immunized mouse spleens. (**A**) IL-17A and IL-4 mRNA expression levels in splenocytes from immunized mice. (**B**) CD4^+^T cells from mice spleens immunized with sE2-mRNA-LNP or sE2_F442NYT_-mRNA-LNP were analyzed for CXCR3 and CCR6 expression. Statistical significance was assessed by one-way ANOVA or Student’s *t*-test and Mann-Whitney *U* test. The significance level is indicated (*P < 0.05; *n* = 5). The percentages of CXCR3^−^ and CCR6^+^ cells in these populations, from five immunized mice, are shown as summarized data, presented as mean ± SD.

We reported earlier the expression of Th1-skewed cytokines from monocyte-derived macrophages of healthy donors by modified sE2_F442NYT_ (13). Incubation with sE2_F442NYT_ generated significantly higher IL-6 levels than unmodified sE2. IL-6 is one of the differentiation factors for the generation of Th17 cells (26). Th17/IL-17 have interesting biological roles, and we became interested in finding if there is a difference in response to the two E2-related immunogens. We did not observe a significant difference in IL-17A induction between sE2_F442NYT_ and sE2-mRNA-LNP immunized mouse spleens (Fig. 3A), while a low level of IL-4 induction was observed from sE2_F442NYT_-mRNA-LNP immunized mice, suggesting a Th2-skewed response by sE2-mRNA-LNP immunization. Gata3, a transcription factor promoting Th2 responses, was significantly lower in sE2_F442NYT_ than in sE2 (Fig. S2). The expression of CXCR3 is preferentially maintained by cells committed to the Th1 pathway, whereas CCR6 is expressed by Th17 cells (27). We examined the population of CXCR3^−^CCR6^+^ cells gated on activation marker CD44 among CD4^+^T cells by flow cytometry (Fig. 3B). The difference in the percentages of CXCR3^−^CCR6^+^ in CD4^+^T cells between sE2 (2.4% ± 1.3%) and sE2_F442NYT_ (2.1% ± 1.5%)-mRNA-LNP immunization was small. These results suggested that both sE2 and sE2_F442NYT_ induce Th17 cells.

### Comparison of CD4 and CXCR5 positive cells induced in mouse spleen following sE2 and sE2_F442NYT_ immunization

Immunization of mice with the modified sE2_F442NYT_-mRNA-LNP generated much improved proinflammatory T cell responses, immunoglobulin class switching, stronger neutralizing antibody response, and improved protective efficacy against surrogate HCV challenge infection, as observed in our previously published studies (13, 15). In contrast, Th17 responses were comparable between the two immunization groups in the present study. The earlier findings on antibody isotype switching and neutralizing antibody responses provided the rationale for investigating potential differences in Th17/IL-17 responses in the current work.

**Fig 4:**
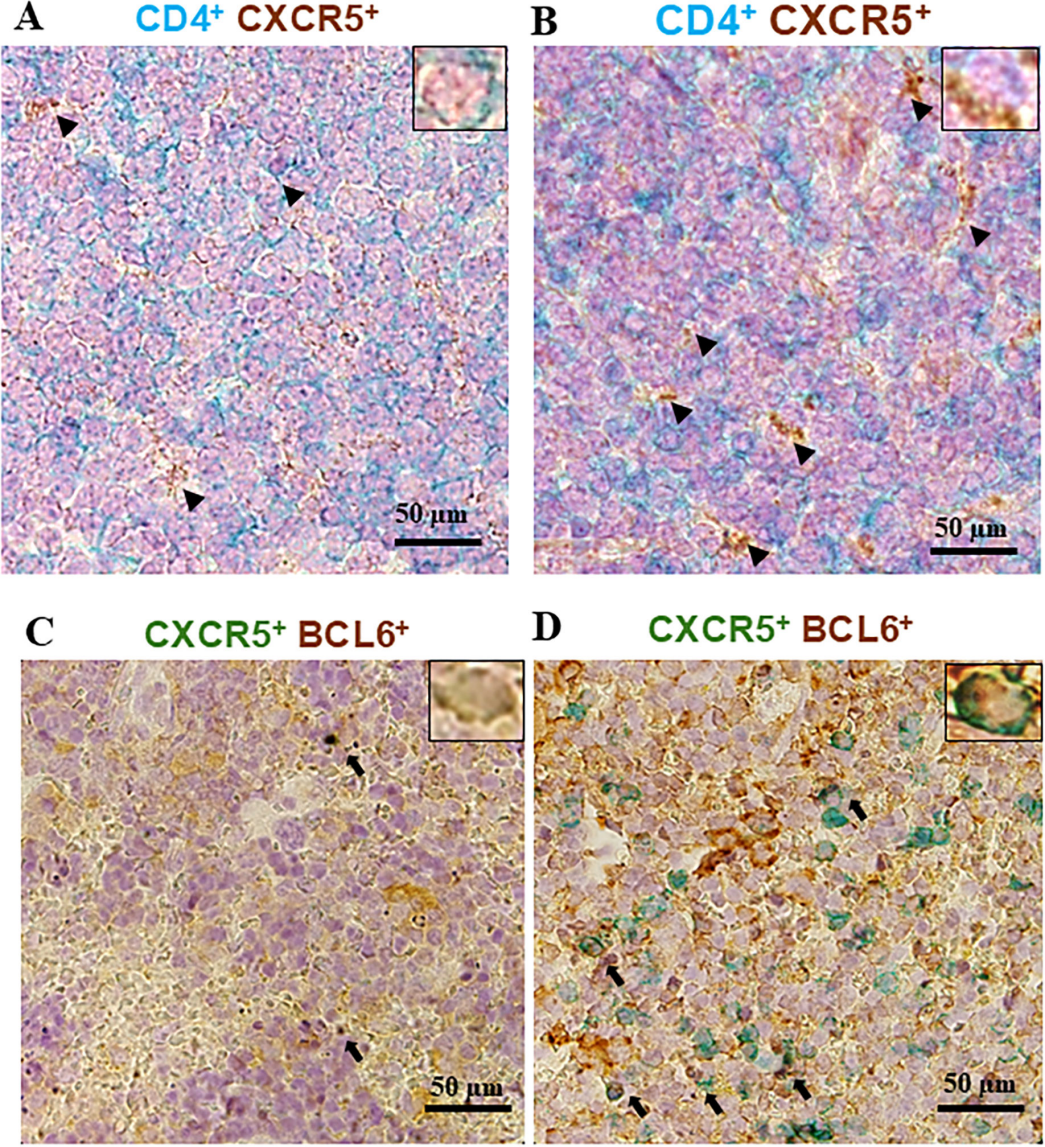
Evaluation of potential T follicular helper (Tfh) cell subsets from sE2 and sE2_F442NYT_ mRNA-LNP immunized mouse spleens. (**A**) Representative immunohistochemical staining of CD4^+^ (blue) and CXCR5^+^ (brown) cells in mouse spleens immunized with sE2-mRNA-LNP or (**B**) sE2_F442NYT_-mRNA-LNP are shown. Black arrowheads indicate CD4^+^CXCR5^+^ double-positive cells. The nuclei were stained with hematoxylin. (C and D) Confocal microscopy results for the association of CXCR5 (green) and BCL6 (brown) in mouse immunized with sE2 or sE2_F442NYT_-mRNA-LNP, respectively, are shown. The results suggested stronger CXCR5^+^ expression in CD4 positive cells (**B**) in association with BCL6 (**D**) in sE2_F442NYT_ immunized mouse spleen as compared to sE2 immunization.

T cells play an important role in initiating antibody response by instructive signals of cell-cell contacts and secretion of soluble cytokines as mediators. The spleen cells and isolated CD4^+^T cells from sE2-mRNA-LNPs or sE2_F442NYT_-mRNA-LNP immunized mice exhibited similar IL-17A levels, and Th17 (CXCR3^−^CCR6^+^) cells in CD4^+^CD44^+^ spleen cells, supporting both sE2 and modified sE2_F442NYT_ induce Th17 polarization. Tfh cells are specialized providers of T cell help to B cells and are essential for germinal center (GC) formation, affinity maturation, development of high-affinity antibodies, and memory and plasma B cells to produce high-affinity antibodies for long-term protective immune response (28). We reported that immunization of mice with sE2_F442NYT_-mRNA-LNP elicited stronger neutralizing antibody response and class switch recombination (15). The high expression of CXCR5 is one of the defining features of Tfh cells, allowing their migration into B cell follicles (24). In our initial examination, we performed double staining of CD4 and CXCR5 for immunohistochemical (IHC) analysis for potential Tfh cells and CXCR5 association with CD4 and BCL6 in mouse spleen immunized with sE2 or sE2_F442NYT_-mRNA-LNP. The representative results are shown (Fig. 4, panels A–D) and suggested higher CXCR5^+^ expression (panel B) in association with BCL6- expressing cells (panels D) especially in sE2_F442NYT_ immunized mouse spleen. These results suggest that sE2_F442NYT_ induces a stronger potential for CXCR5^+^ T cell as a B cell helper function when compared to parental sE2. The stronger B and T cell responses observed from modified sE2_F442NYT_ support the overall *in vivo* outcome of the study toward a higher B helper T cell generation from sE2_F442NYT_-mRNA-LNP immunization as compared to unmodified sE2-mRNA-LNP.

### Immunization with sE2_F442NYT_-mRNA-LNP induces splenic Tfh cells

**Fig 5:**
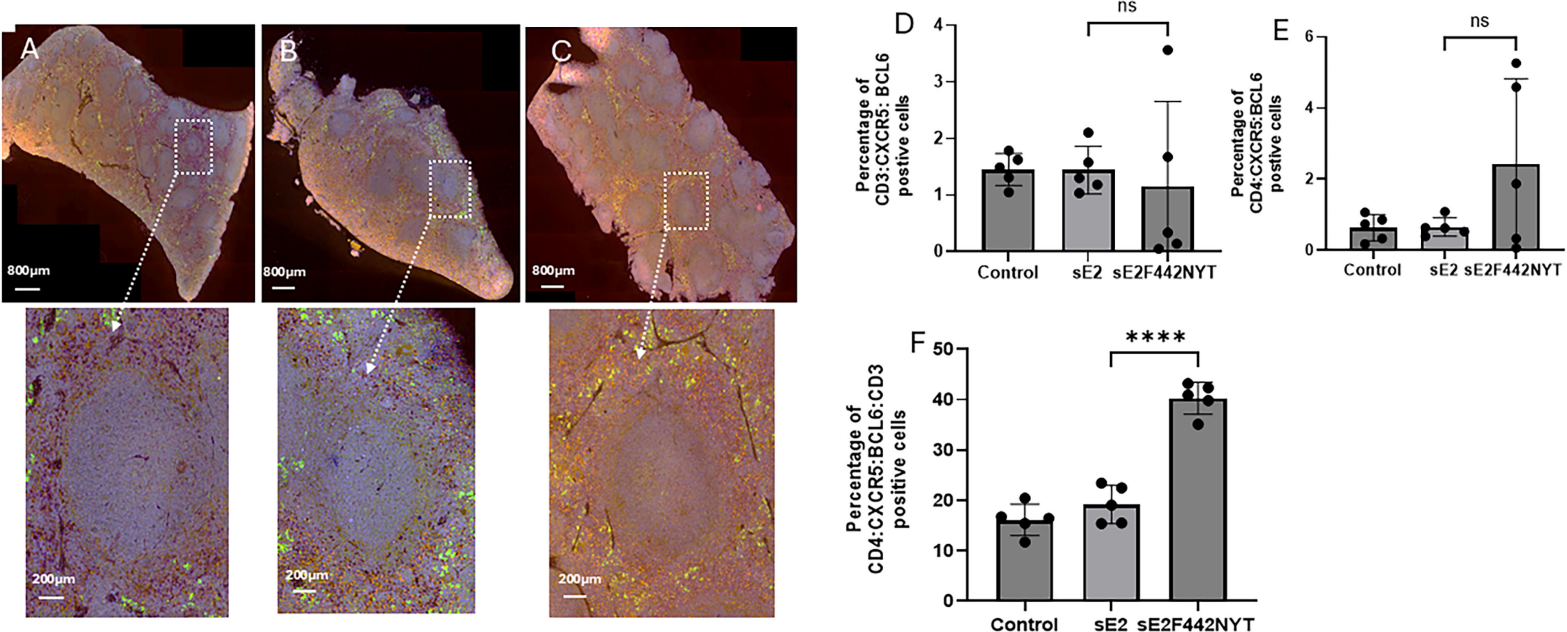
Accumulation of Tfh cells after sE2_F442NYT_-mRNA-LNP vaccination. Selected Confocal enlarged image showing follicular areas from (**A**) empty negative control, (**B**) parental sE2- and (**C**) sE2_F442NYT-_mRNA-LNP vaccinated mouse spleen sections are shown. A zoomed box area with selected CD4 (red), CXCR5 (green), and BCL6 (orange) markers only is shown in the lower panels. A semiquantitative analysis for percentage of marker proteins co-expressed in immune spleen cells is shown at the bottom (**D, E, and F**). Results are presented as mean ± SD. Statistical analysis was performed by one-way ANOVA test (****P < 0.0001).

Previous studies indicated a lack of antibody isotype switching from immunization vaccination with parental E2 in a phase I clinical trial (5, 6) or from immunization vaccination of mice (15). Here, we investigated the induction of Tfh cells in mouse spleen after HCV envelope glycoprotein immunization. We applied multiplex immunofluorescence image analysis allowing for the simultaneous detection of CD4, CD3, CXCR5, and BCL6 in DAPI (nucleus) stained cells (Table 1). The results from imaging data were analyzed from five immunized animals in each group (sE2 immunized: S1–S5, sE2_F442NYT_ immunized: S6–S10, and negative control: S11–S15) on PhenoImager. Our semi-quantitative image analysis suggested higher frequency of CD4 Tfh cells in vaccinated mice compared to empty mRNA-LNP or parental sE2 vaccinated animals. The identification of representative follicular areas expressing CXCR5 and BCL6 marker proteins in experimental mouse spleens is shown (Fig. 5A through C). The percentage of quadruple or triple cell markers is shown (Fig. 5, panels D-F). All four cellular markers were maximum and statistically significant in animals immunized with sE2_F442NYT_ as compared to parental sE2 (panel F). Other marker proteins also showed strong expression although not statistically significant in comparative animal groups (panels D and E). Together, our data suggested that immunization with the modified sE2_F442NYT_ is associated with a significantly higher prevalence of Tfh cells in spleen as compared to the other groups.

**TABLE 1:** Percentage of marker protein co-expressing in immunized spleens by multiplex analysis

Mouse	No. of DAPI positive cells	No. of BCL6:CD3:CXCR5 positive cells	Percentage of BCL6:CD3:CXCR5 positive cells	No. of BCL6:CD4:CXCR5 positive cells	Percentage of BCL6:CD4:CXCR5 positive cells	No. of BCL6:CD4:CXCR5:CD3 positive cells	Percentage of BCL6:CD4:CXCR5:CD3 positive cells
sE2							
S1	10,4297	1,356	1.300133273	408	0.391190542	24,424	23.41773972
S2	45,358	537	1.183914635	240	0.529123859	8,648	19.06609639
S3	64,795	1,023	1.578825527	406	0.626591558	9,966	15.38081642
S4	57,919	599	1.034202939	368	0.635370086	8,995	15.53030957
S5	48,852	1,029	2.106362073	532	1.089003521	10,982	22.48014411
sE2_F442NYT_							
S6	34,500	1,230	3.565217391	21	0.060869565	14,905	43.20289855
S7	63,538	91	0.143221379	2,915	4.587805723	25,306	39.82813434
S8	39,167	132	0.337018408	130	0.331912069	13,746	35.09587152
S9	51,418	862	1.676455716	962	1.870940138	21,767	42.33342409
S10	108,234	56	0.051739749	5,696	5.262671619	44,267	40.89934771
Negative control							
S11	91,374	961	1.051721496	977	1.069231948	18,676	20.43907457
S12	162,438	2,898	1.784065305	543	0.334281387	25,108	15.45697435
S13	139,986	2,082	1.487291586	246	0.175731859	16,393	11.71045676
S14	136,050	2,213	1.626607865	1,166	0.857037854	22,769	16.73575891
S15	132,862	1,744	1.312640183	982	0.739112764	21,850	16.44563532

### sE2_F442NYT_ inhibits natural killer (NK) cell activation

**Fig 6:**
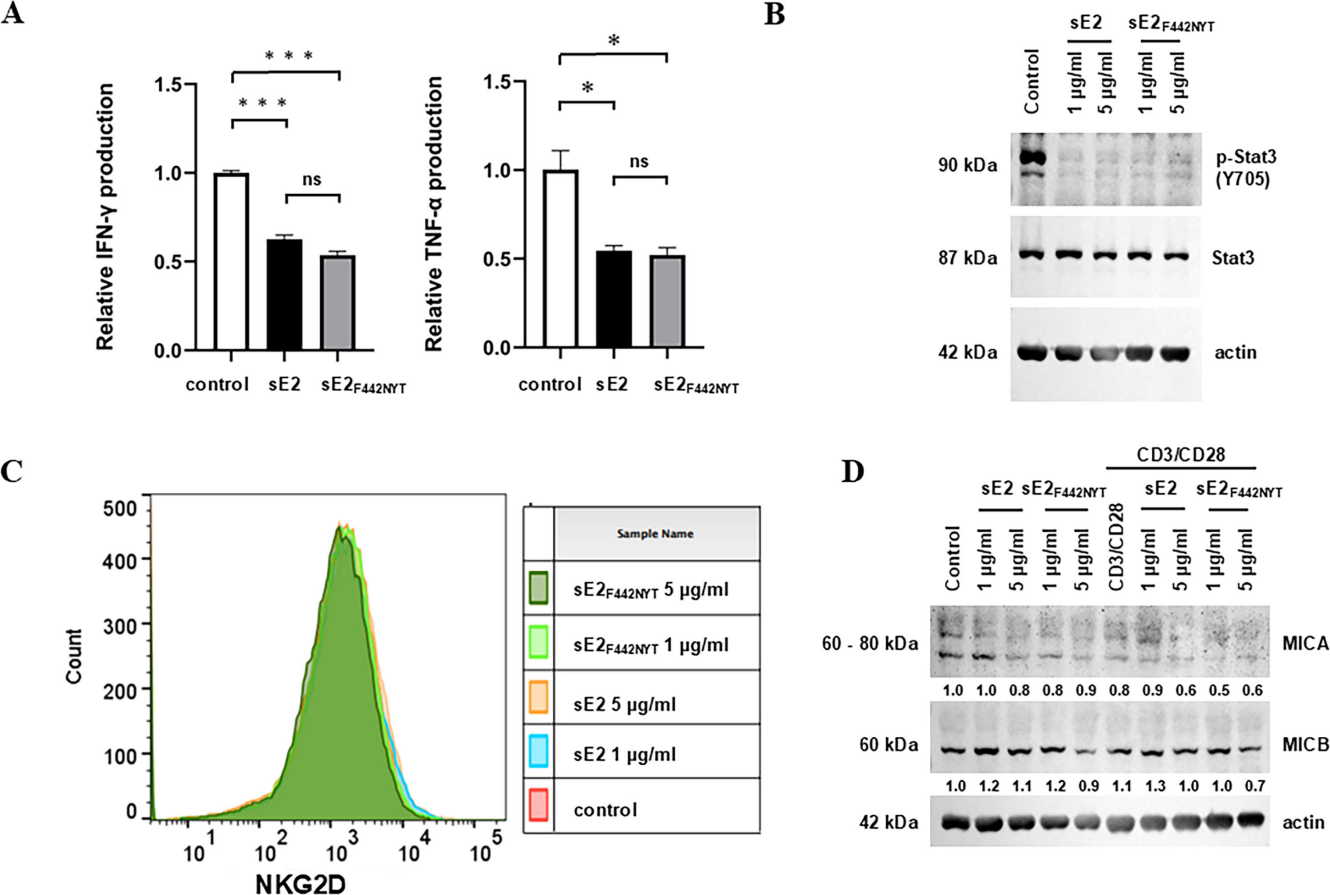
Effect of sE2 and sE2_F442NYT_ on natural killer (NK) cells. (**A**) IFN-γ and TNF-α expression in NK3.3 cells incubated with sE2 or modified sE2_F442NYT_ purified proteins. Data are presented as mean ± SD. Statistical analysis was performed by one-way ANOVA; **P < 0.005, ***P < 0.001. (**B**) Western blot analysis of phosphorylated Stat3 (Y705) and total Stat3 expression in NK3.3 cell lysates. (**C**) Analysis of NKG2D cell surface expression in NK3.3 cells. (**D**) Representative result of Western blot analysis of MICA and MICB expression in human peripheral blood mononuclear cells (PBMCs) from healthy donors is shown.

NK cells are a population of innate immune cells that exhibit potent cytotoxic activity and cytokine production (29). They can affect T cell immunity both directly and indirectly. NK cell-mediated IFN-γ production leads to DC activation. NK cells also promote the differentiation of Tfh cells (30). We examined the effect of sE2 or sE2_F442NYT_ on NK cells. Human NK3.3 cells were cultured in the presence or absence of purified sE2 or sE2_F442NYT_. The level of IFN-γ from NK3.3 cells was suppressed when treated with sE2 compared to untreated control, and sE2_F442NYT_ also inhibited IFN-γ level (Fig. 6A). A similar observation was noted with a change in the TNF-α status. We observed both sE2 and sE2_F442NYT_-inhibited Stat3 phosphorylation in NK3.3 cells compared to untreated control (Fig. 6B), while total Stat3 did not alter significantly, supporting an inhibitory role on NK cell activation.

We analyzed the expression of NK cell activating receptor, the NK group 2D (NKG2D) upon treatment with sE2 and sE2_F442NYT_. The NKG2D expression on NK3.3 cell surface was not significantly decreased upon sE2 or sE2_F442NYT_ exposure at two different concentrations when compared to that of mock-treated NK3.3 cells (Fig. 6C), suggesting none of the antigens showed a major effect on NKG2D expression. The major histocompatibility complex class I chain-related polypeptide A (MICA) and MICB protein expressions were not significantly decreased in sE2 or sE2_F442NYT_ treated human peripheral blood mononuclear cells (PBMCs) (Fig. 6D) irrespective of T cell activation by CD3/CD28 antibodies. Thus, the results suggested that HCV sE2 or sE2_F4422NYT_ inhibited NK cell activation by inhibition of Stat3 phosphorylation regardless of the E2-CD81-binding ability.

## DISCUSSION

**Fig 7:**
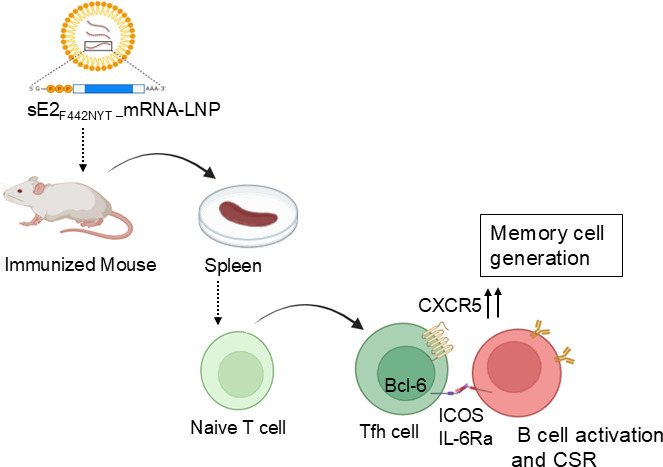
Schematic diagram from the observations suggested that the modified sE2_F442NYT-_mRNA-LNP candidate vaccine induced and detectable CXCR5 for a stronger B helper T cell generation as memory cells (created by BioRender). CSR denotes class switch recombination.

We previously reported that the modified HCV sE2_F442NYT_ envelope glycoprotein dramatically alters cytokine response in mouse or human immune cells and modulates overall adaptive immune responses compared to the unmodified sE2 (13, 15, 16). In this study, we further investigated the interaction of sE2 or sE2_F442NYT_ with DCs, T cells, B cells, and NK cells to understand their immunoregulatory role in assessing candidate vaccine potential. Incubation of human monocyte-derived DCs with purified sE2 or sE2_F442NYT_ induced the production of IL-6 and IL-1β. ERK phosphorylation plays a role in mediating inflammatory signals, and its level negatively correlates with IL-6 expression. Co-culture of autologous human CD4^+^T cells with sE2 or sE2_F442NYT_-exposed DCs resulted in an increased Stat3 phosphorylation, promoting Th17 cell differentiation and IL-17A induction. The spleens of sE2-immunized mouse showed higher mRNA expression of IL-4, IL-17A, Gata3, and Rorc, indicating Th2- or Th17-polarized response, while sE2_F442NYT_ immunization resulted in higher T-bet mRNA expression, suggesting Th1 response. Previous studies showed that ZIKV infection induces Th1-like Tfh cell response during infection or vaccination may augment induction of antiviral antibody response (31). IHC analysis revealed that sE2_F442NYT_ induces a larger population of CXCR5^+^CD4^+^T cells in mouse spleens, a population crucial for Tfh cell differentiation and function (28). Our results show an increase in Tfh cell populations (Fig. 7A), which play a key role in promoting a co-operative B-cell response, particularly with sE2_F442NYT_-mRNA-LNP compared to unmodified sE2, aligning with our previous observations on Ig isotype switching and increased antibody titers (15). IL-6 promotes antibody production by either directly acting on plasma cells or indirectly promoting Tfh cells. This aligns with our findings in the sE2_F442NYT_-immunized mice. On the other hand, IL6 in conjunction with Stat3 and in the presence of TGFβ, IL-1β, or IL-23 promotes differentiation of Th17 cells (32). Additionally, both sE2 and sE2_F442NYT_ similarly inhibited NK cell activation, as evidenced by reduced levels of IFN-γ and TNF-α.

We have shown sE2_F442NYT_ facilitates Th1 type of dominant immune response for protection (17). IL-6 and Th17 are the signature mediators that affect the development of immune dysregulation. IL-6/Stat3, together with IL-1β, induces Th17 cells (33). The differentiation of Th17 cells from naïve CD4^+^T cells is driven by cytokines (34–36). Th17 cells are a proinflammatory subset of CD4^+^T cells characterized by IL-17 production. The sE2/sE2_F442NYT_-treated DCs generated similar levels of these effector cytokines including IL-6 and IL-1β. Consistent with the results from DCs, CD4^+^T cells co-cultured with stimulated DCs generated enhanced levels of IL-17A, suggesting Th17 cells were induced by the sE2 or sE2_F442NYT_. The development of predominant Th17 immunity specific to HCV antigens is linked to suboptimal Th1 responses and increased Th2 immunity to the virus (37). Th17/IL17 responses are interesting for their biological roles, and we were interested in finding if there was a significant difference in response to the immunogens. IL-21-producing Th17 cells were reported to contribute to spontaneous clearance of acute HCV (38), arguing for a protective role of Th17 responses. We became interested in evaluating Th17/IL-17 status in immunized mice with the two different vaccine candidates for immunomodulatory functions. We did not find a significant difference in Th17/IL-17 status, nor in IL-23, for promoting differentiation of Th17 cells between the two groups of immunized mice may be for a difference in the experimental nature and the observations from HCV-infected patient-related response.

We focused on differences in Tfh generation from immunizations with the immunogens in the present study. The proinflammatory T cell responses were similar and modestly higher than that of control. We clarified the previous observations from serum antibody isotype switching and neutralizing antibody response and mentioned as rational at the beginning of the present study. Th17 responses were similar in both the immunization groups and our observations from earlier antibody-related study using sE2_F442NYT_ immunized mice relates with the increase in Tfh cells in the spleen. Further analysis in the future should verify for statistical association of Tfh frequencies in the spleen and will be premature for a conclusion at this time.

Here, the effect of immunization with a lipid-nanoparticle, mRNA delivery system for native, and NYT forms of E1–E2 were examined for their effect on human monocyte-derived DC cells and T cells and also on mouse splenocytes, Tfh cells, and NK cells. Both native and mutant E1–E2 LNP induced IL17 and Stat3 phosphorylation supporting a role in promoting Th17 phenotype. In the mouse spleen cells, CXCR5+T cells co-localized with BCL6 more in those immunized with the NYT mutant, suggesting that this mutant enhances follicular helper T cells.

Th17 cells support antiviral immunity by secreting IL-21, which helps both B cell and CD8+T cell responses in peripheral blood and liver—key elements for effective virus control. The sE2 treatment induced greater Stat3 phosphorylation in CD3/CD28 stimulated CD4^+^T cells when compared to the sE2_F442NYT_ (16). This, along with increased Rorc mRNA expression in splenic tissue and elevated Stat3 phosphorylation in co-cultured CD4^+^T cells suggests that how these two immunogens may promote Th17 differentiation with some degree of variations. Although the functional difference between sE2 and its mutant appears modest, it may be biologically significant, as excessive Th17 responses can also contribute to virus-induced pathology (39).

A key aspect of adaptive immunity to many pathogens and vaccines is the B cell help provided by T cells. Tfh cells are specialized facilitators of B cell help (40). They exhibit dynamic positioning within secondary lymphoid tissues and rely on interactions with APCs for their differentiation and execution of B cell-facilitating functions within germinal centers (41). A precursor subset of Tfh cells is formed upon interaction with APCs, upregulating CXCR5, and migrating to the B-T cell border to interact with cognate B cells. Tfh differentiation begins at the time of APC priming in mice, activating naïve CD4^+^T cells through receiving signals in the form of peptide: MHC, co-stimulatory molecules, and cytokines provided by the APCs. IL-6R and inducible T-cell costimulator (ICOS) are crucial for the full maturation of Tfh cells into germinal center Tfh cells, provide signals for early Tfh differentiation in mice, and contribute to BCL-6 expression (42). BCL-6 plays a central role in controlling Tfh differentiation and function. Stat3 is the most potent inducer of Bcl-6 expression and Tfh differentiation in humans and mice (43). While Tfh differentiation and function are largely conserved between species, there are notable differences in Tfh biology between mice and humans. For instance, IL-6 is a key inducer of Tfh differentiation in mice, whereas activin A is the human Tfh inducer. Further studies demonstrate that HCV-specific CD4^+^T cells with Tfh cell signature are maintained after therapy-induced elimination of persistent infection. Tfh are the important target population for vaccine effort to prevent reinfection (44, 45). Tfh cell frequencies significantly associated with HCV RNA reduction and expansion of memory B cell (46).

In humans, B cells in the GC interact with Tfh cells through which B cells undergo differentiation and proliferation involving a series of maturation steps, including somatic hypermutation, class switching, and affinity maturation, eventually resulting in a population of differentiated plasma cells and memory B cells. There is a lack of isotype switching to IgG2c and IgG2a (31, 47) and of enhanced IgG1 (48) in response to viral infection in the absence of Tfh1 cells. The increase in IgG2a is also consistent with the induction of Th1 cells secreting IFN-γ from HCV sE2_F442NYT_ vaccination (49). Antibody class switching has important functional consequences for lineage maturation and determines the nature of immune cells and molecules recruited by the antibody to help destroy and remove pathogens. A wider role for Tfh1 cells in the GC reaction has been shown in the induction of T-bet in B cells via IFN-γ signaling (50). We examined the presence of Tfh cells in mouse spleens after immunization with the modified sE2_F442NYT_ candidate vaccine by polychromatic flow cytometry, confocal imaging assays. Different molecular signatures and spatial localization within follicular areas may contribute to the heterogeneity of Tfh pool. Our imaging analysis defined follicular areas and facilitated the semi-qualitative analysis of the relevant Tfh cell populations in these areas. Thus, our study illustrated the feasibility of the *in vitro* and *ex vivo* combined approach to dissect the complexity of the human T cell response to HCV E2-derived antigens and reveals novel mechanisms for stable subset differentiation of Tfh cells endowed with different inflammatory capacity as well as factors that modulate their effector functions.

Our future studies on key issues, such as understanding (i) the critical need for the development of a pan-genotypic prophylactic measure against HCV, (ii) the nature of protective immune responses, and (iii) HCV cross-genotype specific protection. Understanding the nature of immune responses generated in non-human primates, resembling those to humans, will facilitate further advancing to a phase 1 clinical trial in humans.

## MATERIALS AND METHODS

### Cells

Human embryonic kidney 293T (HEK293T) cells from American Type Culture Collection (ATCC, Manassas, VA) were maintained in Dulbecco-modified Eagle medium containing 100 U/mL penicillin, 100 µg/mL streptomycin, 10% fetal bovine serum, 1% L-glutamine, and 1% nonessential amino acids (13). The normal human NK cell line, NK3.3 (51), was kindly provided by Jackie Kornbluth (Saint Louis University, MO) and was maintained in RPMI supplemented with penicillin-streptomycin, 20% FCS, 1% L-glutamine, and 125 IU/mL recombinant IL-2 (R & D Systems, Inc., MN). PBMCs were isolated from healthy donor blood (Gulf Coast Regional Blood Center, Houston, TX) using Ficoll-Paque Plus (GE Healthcare) density gradient centrifugation (16). Monocytes and CD4^+^T cells were separated from PBMCs by magnetic beads following the manufacturer’s protocol (Miltenyi Biotec).

### Site-specific mutagenesis of sE2

HCV (genotype 1a/H77C) E2 sequence corresponding to 383–660 amino acids cloned into the pcDNA 3.1 vector (a gift from Heidi E. Drummer) was used as a template for the generation of sE2 mutants using a QuikChange Lightning site-directed mutagenesis kit (Agilent Technology) following the supplier’s procedure. E2 was expressed as C-terminal truncations in mammalian cells since soluble and correctly folded E2 can be obtained by deleting the transmembrane domain (52).

The sE2 double mutant (F442NYT) was generated on the same E2 sequence by replacing two amino acids, as indicated (13). One replacement was done at position 442 with a nonpolar amino acid phenylalanine (F) to a polar amino acid asparagine (N), and another replacement occurred at position 444 of a polar amino acid glutamine (Q) with a polar amino acid threonine (T). The alterations at amino acid positions 442 and 444 (FYQ→NYT) included an N-linked glycosylation site based on an earlier publication (53). The region encompasses a variable sequence and is responsible for inducing interfering antibodies (54). Furthermore, this region may also be involved in inducing antibodies representing a conformational, non-linear epitope including several amino acid residues from distinct locations and abrogates infectivity of H77/JFH1 by substitution at amino acid residue 442 (55). The changes in sE2 with the modification of a glycosylation site (sE2_F442NYT_) were introduced to weaken CD81 binding and, thus, immune cell deregulation for the evaluation of antigenicity in an mRNA-LNP vaccine platform.

Mutations were introduced at positions L427Y, F442N/Y444T, or F442N/Y444T/D535A on sE2 sequence. These amino acids participate in the interaction with CD81 on host cell membrane (53, 56, 57). The specific amino acid changes abrogated the binding of mutant sE2 proteins to CD81 (53). The E2 single and double mutants generated similar ELISA-binding activities with a panel of HCV E2-specific human monoclonal antibodies (CBH2, CBH7, CBH4G, HC-1, HC33.1, and HC-84—gift from Steven Foung, Stanford University, CA) in comparison to the unmodified sE2 protein. These antibodies were from four distinct clusters: CBH2 and HC-1 represent in antigenic domain B (AR3), HC84 on domain D (part of Mansun Law’s AR3), CBH-7 on domain C (AR1), HC33.1 on domain E (AS412). In contrast, our E2 triple mutants lost ~50% ELISA reactivity with CBH2, CBH7, and CBH4G but did not lose reactivity with HC-1, HC-33.1, and HC-84. The introduction of mutations significantly reduced the CD81-binding ability of sE2 mutants compared to the unmodified sE2. We did not include sE2 triple mutants in further experiments since they lost some of the binding activities with humanized mAbs (CBH2, CBH7, and CBH4G) recognizing E2 antigenic sites and could be for changes of antigenic properties.

### qRT-PCR analysis

Total RNA was extracted using TRIzol reagent (Invitrogen, Life Technologies), and cDNA was synthesized with the Superscript III reverse transcriptase kit (Invitrogen) using random hexamers, following the manufacturer’s instructions. Gene expression was quantified using real-time PCR with SYBR Green PCR master mix and detected on an Applied Biosystems 7500 Real-Time PCR System. GAPDH was used as an endogenous control. Relative gene expression was calculated using the 2^−ΔΔCT^ method, where ΔΔCT represents the difference in ΔCT between the sample and control. Primers were purchased from Sigma-Aldrich.

### Generation of mRNA-LNP vaccines encoding HCV envelope glycoproteins

Generation of mRNA vaccines was described previously (13). Codon-optimized HCV (genotype 1a/H77C) envelope glycoproteins sE1 (aa 193–351), sE2 (aa 386–660), and modified sE2_F442NYT_-specific sequence were constructed. HCV sE2 and sE2_F442NYT_ protein samples were examined and compared for the inserted glycosylation site at F442 after PNGaseF treatment at the Washington University Proteomics Shared Resource, St. Louis. The glycosylation site at the residue F442 of modified sE2_F442NYT_ protein was observed following constitutive deamidation of that residue and variable deamidation of other asparagine residues in that sequence. The mRNAs from HCV sE1, sE2, or sE2_F442NYT_ were generated, purified, and encapsulated into LNP for use as candidate vaccines in this study. Codon-optimized HCV E1 (aa193–351) and HCV E2 (aa386–660) specific sequences without or with mutations at residues 442 and 444 were synthesized into mRNAs, purified, and loaded as LNP candidate vaccines formulated for the immunization of mice in this study. The ethanolic lipid mixture comprising an ionizable cationic lipid, phosphatidylcholine, cholesterol, and polyethylene glycol-lipid was rapidly mixed with an aqueous solution at pH 4.0 containing cellulose-purified N1mΨ *in vitro*-transcribed mRNAs. The ionizable lipid and LNP formulation used in this study is proprietary to Acuitas Therapeutics (US patent 10, 221, 27). RNA-loaded particles were characterized for their size, surface charge, encapsulation efficiency, and endotoxin content and subsequently stored at −80°C at an RNA concentration of 1  µg/µL or loaded particles or an equivalent total lipid concentration for empty particles (58). The mean hydrodynamic diameter of mRNA-LNP was ~80  nm, with a polydispersity index of 0.02–0.06 and an encapsulation efficiency of ~95%. Two or three batches from each mRNA-LNP formulations were used in these studies.

### Cytokine quantification from antigen-presenting cells and Western blot analysis

For the generation of DCs, monocytes were maintained with granulocyte-macrophage colony-stimulating factor (GM-CSF, Sino Biological) and IL-4 (Millipore, Sigma) for 7 days (16). Monocyte-derived DCs were stimulated with sE2 or modified sE2_F442NYT_ for 24  h. Culture supernatant and cells were harvested. Cytokine levels were measured by ELISA (IL-6: Sino Biological; IL-1β: Biolegend; IL-23: Thermo Scientific). Protein expression and phosphorylation were determined by western blot analysis as previously described (12).

### Antigen-presenting cells/CD4^+^T cell co-culture

Generated DCs were stimulated with sE2 or modified sE2_F442NYT_ for 24  h. Autologous CD4^+^T cells and antigen-presenting cells (10:1) were cocultured for 7  days, and then culture supernatant and cells were harvested. Cytokine levels (IL-17A) were measured by ELISA (Sino Biological)

### Immunization of mice with mRNA-LNP vaccine

BALB/c mice (Jackson Lab) were divided into groups of five mice each and immunized intra-muscularly with 10  µg mRNA-LNP candidate vaccines, either sE2 or sE2_F442NYT_, administered twice at 2 week intervals. Control mice were immunized with empty-mRNA-LNP. Mice were sacrificed 2 weeks after the second immunization for the collection of the spleens for further analysis. All animal experiments were performed in accordance with NIH guidelines following a protocol approved (protocol no. 2891) by the Institutional Animal Care and Use Committee (IACUC) of Saint Louis University.

### Immunohistochemistry

Mice spleens were harvested and fixed in 10% formalin. Antigen retrieval on mouse-derived tissues was performed by steaming samples in citrate buffer for 20 min before staining with antibodies for CXCR5 (AB254415-1001, used at 1:200 dilution), CD4 (AB183685-1001, used at 1:400), and a secondary goat anti-rabbit IgG H and L HRP (AB 6721-1001) according to the manufacturer’s recommendation (Abcam). IHC staining was counterstained with hematoxylin to detect nuclear proteins and photographed (Nikon). The quantification of double-positive cells was validated in a Nikon Eclipse Ni microscope. Cells were also stained separately with antibodies to CXCR5 and BCL6 and photographed using ECHO REVOLVE Microscope imaging system.

### Multiplex confocal imaging of immune spleen sections

Spleen tissue from at least three differently randomly chosen sections was processed as described earlier (58, 59). Slides were baked at 65°C for 30 min, dewaxed, rehydrated, and antigen-retrieved (Tris-EDTA, pH 9) using the Parhelia Spatial Station. Slides were washed with PBS and Akoya staining buffer (PBS containing 0.5% BSA) and blocked with Akoya Blocking solution for 30 min at room temperature before staining by antibodies (Table 2). Slides were incubated with rabbit anti-mouse CXCR5 (1:100) overnight at 4°C in a humid chamber. The next day, they were washed four times with PBS, followed by three times with PBS containing 0.1% Tween 20. Donkey anti-rabbit-AF488 (1:150) was applied for 45 min at room temperature in a dark humid chamber. Slides were washed with PBS containing Tween20. Next, rabbit anti-CD4-AF647 (1:150) was added and incubated for 30 min at room temperature to bind the CD4 receptor prior to adding anti-CD3.A cocktail of the following prepared antibodies in Akoya staining buffer prepared applied to each slide (120 µL): Rat anti-mouse CD3-AF750 (1:100), Rabbit anti-mouse CD4-AF647 (1:65), Rabbit anti-BCL6-AF594 (1:100). Slides were incubated overnight at 4°C in a dark humid chamber and washed with PBS followed by PBS containing 0.1% Tween 20.

Rabbit anti-rat IgG (H&L)-biotinylated (1:100) in PBS containing 0.1% Tween 20 was added and incubated for 45 min at room temperature in the dark. Slides were washed with PBS containing 0.1% Tween 20. Antibody details are provided in Table 2.

**TABLE 2:** Antibodies used

Antibody	Vendor	Catalog no.
Anti-mouse CD4 polyclonal AF647	Novus	NBP3-18057AF647
Anti-mouse BCL6 (IG191E/A8) AF594	Biolegend	648308
Rb anti-mouse CXCR5 (JB11-40) unconj R&D Systems	Novus	NBP2-75460
Donkey anti-rabbit IgG AF488 (min x reactivity)	Biolegend	406416
Rat anti-mouse CD3 (17A2) AF750	R&D Systems	FAB4841RS10
Rb anti-rat IgG H&L biotinylated	Vector Lab	BA-4001
SA-PE 0.2 mg/mL	Biolegend PE	–^*a*^

^*a*^ –, not applicable.

Streptavidin PE (1:300) in PBS containing 0.05% Tween 20) was added and incubated for 1 h at room temperature in the dark. Slides were washed with PBS containing 0.1% Tween 20 and then PBS. Akoya DAPI (diluted 1:180 in PBS, 120 µL) was applied and incubated for 5 min, washed with PBS, and then mounted with Prolong Gold. Imaging was performed on PhenoImager using unmixing to remove autofluorescence. PhenoImager allowed high-resolution, high-throughput spatial biology crucial for spatial distribution of biomolecules in tissue. Opal colors associated with the panel were analyzed as follows: Opal 520: CXCR5 Opal 570: CD3 (PE using anti-rat IgG), Opal 620: BCL6, Opal 690: CD4, Opal 780: CD3 AF750. CD4 T cell staining was not very strong as anticipated although CD3 staining as a marker for T cells was very prominent. In our experiment, CD4^+^ color intensity in cells was weak in general and that might have contributed to statistical variations in data sets. However, the results from CD3 staining compensated for the weakness in CD4^+^ cell marker expression in data interpretation.

Imaging data sets were segmented post-acquisition based on their nuclear staining signal, and average voxel intensities for all channels were extrapolated in Imaris after iso-surface generation. Data were exported to Microsoft Excel, concatenated into a single comma-separated values format, and imported into FlowJo version 10 for further analysis. All these steps and the processes were performed at the Immunomonitoring Laboratory Facility, Washington University, Saint Louis. The image analysis was processed in software QuPath 0.5.1 (60). Each tissue was annotated into two sections, and cell detection was performed following single measurement classifier.

### Statistical analysis

All data were analyzed using GraphPad Prism 10 software. Statistical analysis with two groups was performed using a two-tailed unpaired student *t* test or Mann-Whitney *U* test. One-way ANOVA test was used for multiple pairwise comparisons between groups. A *P* value of < 0.05 was considered significant.

## Data Availability

All data underlying the results are available as part of the article, and no additional source data are required.

## SUPPLEMENTAL MATERIAL

Supplemental figures jvi.01355-25-s0001.pdf Figures S1 and S2.

ASM does not own the copyrights to Supplemental Material that may be linked to, or accessed through, an article. The authors have granted ASM a non-exclusive, world-wide license to publish the Supplemental Material files. Please contact the corresponding author directly for reuse.
