# Supplementary material for: Hepatitis C virus modified SE2F442NYT-mRNA-LNP candidate vaccine promotes helper CXCR5+T cells
Source: J Virol. 2025 Sep 5;99(10):e01355-25. doi: 10.1128/jvi.01355-25 (PMC12548457; doi:10.1128/jvi.01355-25)
Supplement: Supplemental figures — Figures S1 and S2. [file jvi.01355-25-s0001.pdf]

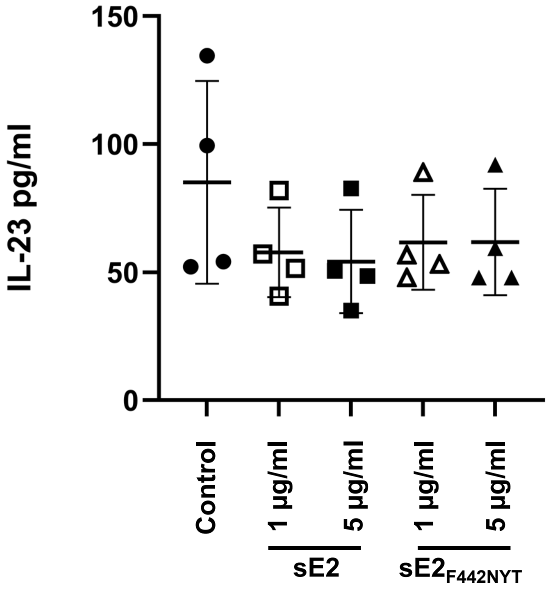

Supplementary Fig. 1

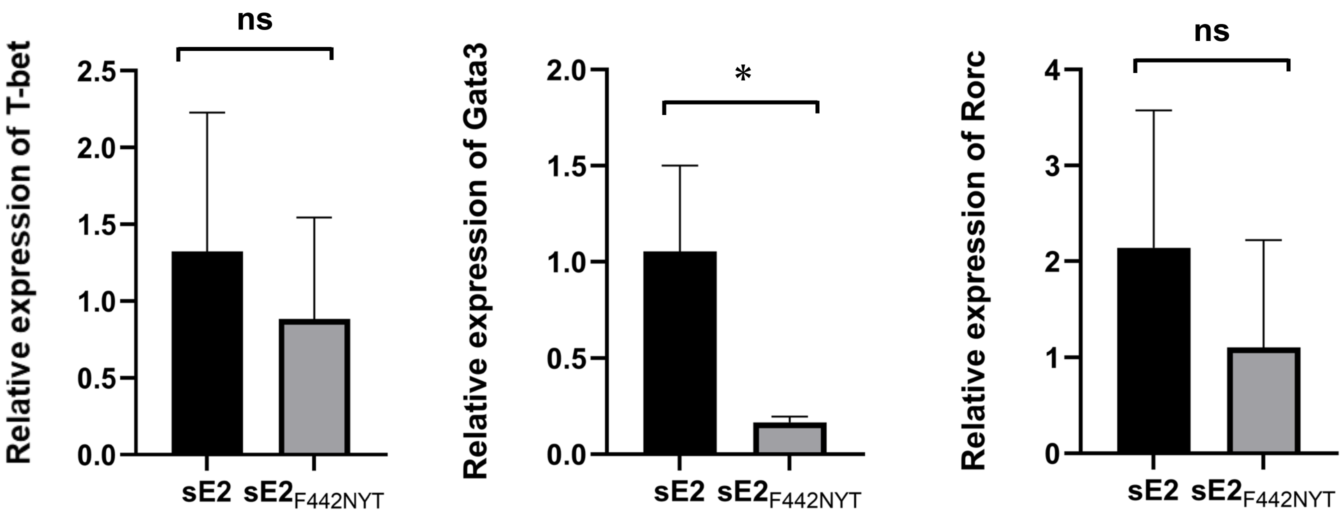

Supplementary Fig. 2

Supplementary Fig. 1. IL-23 generation from monocyte-derived DCs. Expression of IL-23 from monocyte-derived DCs incubated with sE2 or modified $sE2_{F442NYT}$ purified proteins. Data are presented as mean ± SD. Statistical analysis was performed by one-way ANOVA test.

Supplementary Fig. 2. Transcription factors for Th cell differentiation in sE2 and $sE2_{F442NYT}$-mRNA-LNP immunized mouse spleen. T-bet, Gata3, and Rorc mRNA expression in splenocytes from mice spleen immunized by sE2- or $sE2_{F442NYT}$-mRNA-LNP. Bars represent mean values with SD. Statistical analysis was performed by one-way ANOVA test. The significance levels are indicated (*$p < 0.05$; $n = 5$).
